# 16S ribosomal ribonucleic acid sequencing reveals bile microbiome features in gallstone disease and their links to blood lipid subtypes

**DOI:** 10.3389/fmicb.2026.1741489

**Published:** 2026-02-16

**Authors:** Shijie Wang, Yongxing Ding, Cheng Cai, Mingrui Ou, Chunshen Niu

**Affiliations:** 1 Department of Hepatobiliary Surgery, Third People's Hospital of Bengbu City, Bengbu, Anhui, China; 2 Department of Surgery, Bengbu Medical University (Residency Training Base of Surgery), Third People's Hospital of Bengbu City, Bengbu, Anhui, China

**Keywords:** bile, bile acids and salts, gallstones, hypertriglyceridaemia, microbiota

## Abstract

**Background:**

Gallstone disease (GSD) represents a major global health burden with complex pathophysiology involving bile microbiome dysbiosis and metabolic dysfunction. Although previous studies have examined bile microbial communities, the relationship between bile microbiome composition and specific lipid phenotypes remains incompletely understood.

**Methods:**

We conducted a cross-sectional study of 28 adults undergoing cholecystectomy for radiologically and pathologically confirmed gallstones. Bile samples were collected intraoperatively and subjected to 16S ribosomal ribonucleic acid V3–V4 region sequencing. Patients were stratified by lipid subtypes based on contemporary dyslipidaemia guidelines. Microbial diversity, community structure and differential abundance analyses were performed alongside machine learning classification.

**Results:**

The bile microbiome exhibited distinct compositional patterns between the hypertriglyceridaemia (HTG) and non-HTG (NTG) groups, with key phyla (Proteobacteria, Firmicutes) showing group-specific abundance trends and alpha diversity indices reflecting reduced evenness in HTG. Beta diversity analyses demonstrated mild-to-moderate separation between groups, and the linear discriminant analysis effect size technique identified discriminatory taxa with potential functional relevance. Random forest classification achieved moderate accuracy in predicting lipid subtypes based on microbial features.

**Conclusions:**

This study revealed associations between bile microbiome composition and systemic lipid metabolism in GSD, suggesting potential mechanistic links through bile acid metabolism and farnesoid X receptor–fibroblast growth factor 19 signaling pathways.

## 1 Introduction

Gallstone disease (GSD) constitutes one of the most prevalent digestive disorders worldwide, affecting approximately 10%−15% of adults in developed countries and imposing substantial healthcare costs (Lammert et al., 2016). The pathogenesis of gallstones involves complex interactions between genetic predisposition, metabolic dysfunction and environmental factors, with emerging evidence highlighting the critical role of the biliary microbiome in disease development (Chen et al., 2022). Recent advances in high-throughput sequencing technologies have revolutionized the understanding of microbial communities within the biliary system, challenging the long-held paradigm that bile represents a sterile environment (Molinero et al., 2019).

The discovery of microbial communities within the gallbladder has opened new avenues for understanding gallstone pathophysiology. The main phyla found in bile were Firmicutes, Bacteroidetes, Actinobacteria and Proteobacteria (Molinero et al., 2019). These findings have been consistently replicated across multiple populations, suggesting a conserved biliary microbiome structure that may be disrupted in disease states (Shen et al., 2015). The composition of the bile microbiome appears to be influenced by multiple factors, including diet, metabolic status and genetic variation, with significant implications for gallstone formation (Wu et al., 2013).

Metabolic syndrome and dyslipidaemia have been increasingly recognized as important risk factors for gallstone development. Multiple studies have investigated the associations between blood lipid metabolism and GSD risk; however, the results are inconsistent (Zhang et al., 2022). The relationship between lipid metabolism and gallstone formation involves complex mechanisms, including altered cholesterol homeostasis, impaired bile acid synthesis and dysregulated enterohepatic circulation (Wang and Cohen, 2020). Recent evidence suggests that specific lipid phenotypes may predispose individuals to distinct patterns of bile microbiome dysbiosis, potentially contributing to stone formation through multiple pathogenic pathways (Grigor'eva and Romanova, 2020). Notably, dyslipidaemia, particularly elevated triglycerides (TGs), can directly modulate the bile microbial community structure by altering the bile acid composition and biliary microenvironment, creating a bidirectional interaction that may amplify metabolic dysfunction and gallstone risk.

The farnesoid X receptor–fibroblast growth factor (FGF) 19 signaling axis represents a critical regulatory mechanism linking bile acid metabolism, lipid homeostasis and gut microbiota (Jia et al., 2018). At the molecular level, *Lactobacillus reuteri* and *L. plantarum* increased ileal FGF 15 and hepatic FGF receptor 4 and small heterodimer partner (Ye et al., 2022). This signaling pathway modulates bile acid synthesis through feedback inhibition of CYP7A1, the rate-limiting enzyme in bile acid production, and influences gallbladder motility and bile composition (Kong et al., 2012).

The gut–liver–gallbladder axis has emerged as a crucial determinant of gallstone susceptibility. The microbiome of bile correlates with the bacterial composition of saliva, and the microbiome of the biliary tract has a high similarity with the microbiota of the duodenum (Fang et al., 2024). This anatomical and functional connectivity suggests that perturbations in one compartment may have cascading effects throughout the system, potentially explaining the systemic metabolic disturbances observed in patients with gallstones (He et al., 2020).

Recent technological advances have enabled more sophisticated analyses of microbiome–host interactions. Machine learning and deep learning approaches have been increasingly applied to microbiome research, allowing for the identification of complex microbial signatures associated with disease states (Namkung, 2020). These computational approaches have revealed previously unrecognized patterns in microbiome data that may serve as biomarkers for disease risk stratification and therapeutic targeting (Topçuoglu et al., 2020).

The clinical significance of understanding bile microbiome–lipid metabolism interactions extends beyond academic interest. The abundance of bacteria in patients with GSD significantly increased, whereas the diversity of bacteria decreased (Lv et al., 2021). These alterations in microbial community structure may contribute to the chronic inflammation and metabolic dysfunction observed in GSD, suggesting potential therapeutic targets for disease prevention and management (Hu et al., 2022). Chronic inflammation in the biliary tract, such as that seen in immune-mediated cholangitis induced by checkpoint inhibitors, such as nivolumab and pembrolizumab, can further disrupt microbial homeostasis and lipid metabolism, highlighting the interconnectedness of immune responses, biliary inflammation and metabolic phenotypes (Fang et al., 2024; He et al., 2020).

Despite growing interest in the bile microbiome, several knowledge gaps remain. The temporal dynamics of microbial colonization, the influence of specific dietary components on bile microbiome composition and the potential for microbiome-based interventions in gallstone prevention require further investigation (Chen et al., 2019). Additionally, the sex-specific differences in gallstone prevalence and the potential role of hormonal factors in modulating bile microbiome–lipid interactions warrant detailed examination (Di Ciaula et al., 2019).

Emerging evidence indicates that the gut–brain axis may also modulate lipid metabolism through microbial pathways, as demonstrated in studies of olanzapine-induced lipid disturbances, suggesting that systemic metabolic effects of microbiota extend beyond the gut–liver axis to other organ systems (Zhu et al., 2022). The present study aims to characterize comprehensively the bile microbiome in patients with gallstones using 16S ribosomal ribonucleic acid (rRNA) sequencing and to investigate associations between microbial community composition and blood lipid phenotypes. By integrating microbiome profiling with detailed metabolic phenotyping, the study seeks to identify potential mechanistic links between bile dysbiosis and systemic lipid metabolism that may inform future therapeutic strategies for GSD prevention and management.

## 2 Materials and methods

### 2.1 Study population and sampling

This cross-sectional study was conducted between July 2022 and September 2023 at Bengbu Third People's Hospital following institutional review board approval. A total of 28 adult patients (aged 29–69 years) scheduled for elective cholecystectomy with radiologically confirmed gallbladder stones were prospectively enrolled. The diagnosis of GSD was established through ultrasonographic examination demonstrating hyperechoic structures with acoustic shadowing within the gallbladder lumen, subsequently confirmed by pathological examination of surgical specimens. The exclusion criteria were as follows: (1) current or recent antibiotic therapy within 30 days prior to surgery; (2) presence of acute cholangitis or severe cholecystitis requiring emergency intervention; (3) documented inflammatory bowel disease or other chronic gastrointestinal disorders; (4) active malignancy or immunosuppressive therapy; (5) pregnancy or lactation; or (6) inability to provide informed consent.

Intraoperative bile collection was performed using standardized aseptic techniques to minimize contamination. Following gallbladder exposure, but before dissection from the hepatic bed, the surgeon performed direct aspiration of bile using an 18-gauge needle attached to a sterile syringe. Approximately 10–15 ml of bile was collected from each patient, immediately transferred to sterile cryovials, placed on ice and transported to the laboratory within 30 min of collection. Samples were aliquoted to avoid repeated freeze–thaw cycles and stored at −80 °C until deoxyribonucleic acid (DNA) extraction. Comprehensive clinical data were collected through structured questionnaires and electronic medical record review, including demographic characteristics [age, sex, and body mass index (BMI)], medical history (comorbidities, medication use), laboratory parameters [complete blood count and liver function tests, including alanine aminotransferase (ALT), aspartate aminotransferase (AST), gamma-glutamyl transferase (GGT), bilirubin levels, fasting glucose, C-reactive protein (CRP), total bile acids], imaging findings (gallbladder wall thickness, stone number and size, presence of fatty liver). The pathological examination of resected gallbladders was performed by experienced pathologists blinded to the microbiome analysis results.

### 2.2 Serum lipid phenotyping

Fasting venous blood samples were collected within 24 h prior to surgery following an overnight fast of ≥12 h. Serum lipid profiles were determined using automated biochemical analysers (Roche Cobas 8000 series; Roche Diagnostics GmbH, Mannheim, Baden–Württemberg, Germany; authorized local distribution in China: Roche Diagnostics Products (Shanghai) Co., Ltd., Shanghai, Shanghai Municipality, China) with standardized enzymatic methods. Total cholesterol (TC) was measured using the cholesterol oxidase–peroxidase method, TGs by the glycerol phosphate oxidase–peroxidase method, high-density lipoprotein cholesterol (HDL-C) by direct homogeneous enzymatic assay and low-density lipoprotein cholesterol (LDL-C) was calculated using the Friedewald equation when TGs were < 400 mg/dl or measured directly when TGs exceeded this threshold.

For the primary analysis, patients were stratified into two groups based on TG levels according to contemporary dyslipidaemia guidelines: (1) a hypertriglyceridaemia (HTG) group, defined as fasting TGs ≥1.7 mmol/L (150 mg/dl); and (2) a non-HTG (NTG) group, with TGs < 1.7 mmol/L. This binary classification was chosen to focus on the relationship between elevated TGs—a key component of metabolic syndrome strongly associated with gallstone formation—and bile microbiome composition. The TG threshold of 1.7 mmol/L represents the internationally accepted cutoff for defining HTG and increased cardiovascular risk. This stratification approach served as the primary grouping variable for all subsequent microbiome comparative analyses.

### 2.3 Deoxyribonucleic acid extraction and 16S library preparation

Microbial DNA extraction from bile samples was performed using a modified protocol optimized for low-biomass samples with high concentrations of bile salts and other inhibitory compounds. Extraction blanks (no sample input) were included as negative controls to monitor potential contamination during DNA extraction and library preparation. Bile samples (200 μl) were first treated with mutanolysin (25 U/ml) and lysozyme (20 mg/ml) for 1 h at 37 °C to enhance bacterial cell wall disruption. Subsequently, mechanical lysis was achieved through bead-beating using 0.1 mm zirconia/silica beads (BioSpec Products) in a FastPrep-24 homogeniser (MP Biomedicals) at 6.0 m/s for 60 s, repeated twice with 5-min cooling intervals on ice. Extraction of DNA then proceeded using the QIAamp DNA Mini Kit (Qiagen) with modifications including extended proteinase K digestion (2 h at 56 °C) and addition of carrier RNA to improve DNA recovery from low-biomass samples.

The V3–V4 hypervariable region of the bacterial 16S rRNA gene was targeted for amplification using universal primers 341F 5′-CCTAYGGGRBGCASCAG3′) and 806R 5′-GGACTACNNGGGTATCTAAT3′) containing Illumina adapter sequences and sample-specific barcodes. Polymerase chain reaction (PCR) amplification was performed in triplicate 25 μl reactions containing 12.5 μl 2 × KAPA HiFi HotStart ReadyMix, 0.5 μM of each primer and 5 ng template DNA. Thermal cycling conditions consisted of initial denaturation at 95 °C for 3 min, followed by 30 cycles of 95 °C for 30 s, 55 °C for 30 s and 72 °C for 30 s, with final extension at 72 °C for 5 min. Triplicate PCR products were pooled, purified using AMPure XP beads (Beckman Coulter) and quantified using Qubit fluorometry and quantitative PCR (qPCR).

Library preparation followed the NEBNext Ultra II DNA Library Prep Kit protocol with size selection targeting 450–550 bp fragments. Final libraries were validated using the Agilent 2100 Bioanalyzer for size distribution and molarity calculation. Equimolar pooling of indexed libraries was performed based on qPCR quantification, with a 20% PhiX control spike-in to increase sequence diversity. Paired-end sequencing (2 × 250 bp) was conducted on the Illumina MiSeq platform following the manufacturer's protocols, generating approximately 100,000 reads per sample to ensure adequate coverage for rare taxa detection.

### 2.4 Bioinformatics

The bioinformatics pipeline integrated multiple quality control and analysis steps to ensure robust taxonomic profiling and diversity assessment. (1) Quality control and read processing began with raw sequence demultiplexing using bcl2fastq, followed by adapter and primer removal using cutadapt v3.5 with error tolerance of 0.15. Quality filtering was performed using fastp v0.23 with parameters including minimum quality score of Q20, minimum read length of 200 bp and sliding window trimming (window size = 4, quality threshold = 20). Paired-end reads were merged using USEARCH v11 with a minimum overlap of 16 bp and a maximum mismatch ratio of 0.1. (2) Amplicon sequence variant (ASV) inference employed the UNOISE3 algorithm for denoising, which simultaneously performs error correction and chimera removal. This approach generates exact sequence variants providing single-nucleotide resolution superior to traditional operational taxonomic unit (OTU) clustering. Additional chimera filtering was performed using UCHIME in reference mode against the SILVA database. Sequences identified as chloroplast, mitochondrial or unclassified at the kingdom level were removed from further analysis. Data preprocessing steps were implemented in accordance with established best practices to minimize bias and ensure data quality, as highlighted by He et al. (2020). (3) Taxonomic assignment utilized the SILVA v138.1 reference database with the Ribosomal Database Project Classifier algorithm at a 0.8 confidence threshold. This threshold balanced sensitivity and specificity for bile samples, which often contain taxa absent from reference databases. The phylogenetic tree construction for the unique fraction metric (UniFrac) analyses employed MUSCLE alignment followed by FastTree. (4) Diversity metrics were calculated after rarefaction to 10,000 sequences per sample to standardize the sequencing depth. All 28 samples were retained after rarefaction, with no samples excluded due to insufficient read depth. The alpha diversity indices included observed ASVs (richness), Chao1 (estimated richness), Shannon entropy (evenness and richness), Simpson index (dominance) and Pielou's evenness. Beta diversity was assessed using multiple distance metrics, including Bray–Curtis (abundance-based), Jaccard (presence/absence) and weighted/unweighted UniFrac (phylogenetically-informed). Ordination techniques included non-metric multidimensional scaling (NMDS) with stress assessment, principal component analysis (PCA) and principal coordinates analysis (PCoA). (5) Differential abundance testing employed multiple complementary approaches. Non-parametric tests (Wilcoxon rank-sum for pairwise comparisons, Kruskal–Wallis for multi-group) were applied with the Benjamini–Hochberg false discovery rate (FDR) correction at *q* < 0.05. The linear discriminant analysis effect size (LEfSe) technique was performed using the default parameters in the Galaxy platform. The LEfSe identified biomarkers with a linear discriminant analysis score threshold of 2.0, incorporating both statistical significance (Kruskal–Wallis, *p* < 0.05) and biological relevance (effect size). (6) Functional prediction utilized PICRUSt2 for inferring metabolic pathways from 16S data, mapping to the Kyoto Encyclopedia of Genes and Genomes and MetaCyc databases. Random forest classification was implemented using 1,000 trees with fivefold cross-validation for feature importance ranking. The random forest models were constructed using the randomForest package in R, with model performance evaluated using precision, recall and area under the receiver operating characteristic curve (AUC-ROC) in addition to the out-of-bag (OOB) error rate. Feature importance was quantified using two metrics: mean decrease accuracy (reduction in classification accuracy when the feature is permuted) and mean decrease Gini (reduction in Gini impurity when the feature is used for splitting nodes in the decision trees), both of which reflect the contribution of each OTU in distinguishing between the HTG and NTG groups.

### 2.5 Statistical considerations

Statistical analyses were performed using R v4.2.0 with specialized packages for microbiome analysis, including the phyloseq, vegan and microbiome packages. Continuous variables were assessed for normality using Shapiro–Wilk tests and presented as mean ± standard deviation for normally distributed data or median with interquartile range (IQR) for non-parametric data. Categorical variables were expressed as frequencies and percentages. Between-group comparisons employed Student's *t*-test or Mann–Whitney *U* test for continuous variables and the chi-squared or Fisher's exact test for categorical variables, as appropriate.

For microbiome-specific analyses, community structure differences were evaluated using permutational multivariate analysis of variance (PERMANOVA/Adonis) with 9,999 permutations and analysis of similarities (ANOSIM) to test for significant clustering by lipid phenotype. The PERMANOVA was performed using the adonis2 function in the vegan package, with Bray–Curtis distances as the input. The dispersion homogeneity was verified using PERMDISP to ensure the PERMANOVA assumptions were met. Multiple testing correction was applied throughout using the Benjamini–Hochberg method with an FDR threshold of 0.05. Effect sizes were calculated using Cohen's *d* for parametric comparisons and Cliff's delta for non-parametric tests.

A *post-hoc* power assessment indicated that the sample size of 28 patients provided 75% power to detect a moderate effect size (Cohen's *d* = 0.8) in the Simpson diversity index (the primary diversity outcome) between HTG and NTG groups at alpha = 0.05.

Given the small sample size (*n* = 28) and potential for overfitting, the model performance was evaluated using fivefold cross-validation, and the overfitting risk was assessed by comparing training and cross-validation error rates. No evidence of significant overfitting was observed (training error rate: 21.4%; cross-validation error rate: 32.1%). Additionally, the study population size is relatively modest, which may limit generalisability to larger cohorts; however, this sample size is consistent with similar low-biomass microbiome studies in biliary samples (Grigor'eva and Romanova, 2020; Fang et al., 2024).

The study cohort exhibited a significant gender imbalance (85.7% women), which was acknowledged as a potential source of bias. This gender distribution aligns with epidemiological patterns of GSD (women have 2–3 times higher prevalence than men) but may limit the generalisability of findings to male populations. Sensitivity analyses excluding male patients (*n* = 4) did not substantially alter the primary findings, suggesting that gender imbalance did not majorly confound the observed microbiome–lipid associations.

### 2.6 Ethics

This study was conducted in accordance with the Declaration of Helsinki and received approval from the institutional review boards of all participating centers. Written informed consent was obtained from all participants prior to enrolment, with specific consent for bile sample collection and genetic/microbiome analyses. Patient confidentiality was maintained through de-identification of all samples and data, with unique study identifiers assigned to each participant. Biospecimens were handled according to international guidelines for human biological material, with appropriate biosafety measures for potentially infectious samples.

## 3 Results

### 3.1 Clinical characteristics

The study cohort comprised 28 patients with confirmed GSD and demonstrated a predominance of women (85.7%, *n* = 24); this is consistent with established epidemiological patterns, where women have 2–3 times higher gallstone prevalence than men, attributed to estrogen effects on cholesterol metabolism and gallbladder motility. The median age was 53.5 years (IQR 40.75–58.75, range 29–69), with a mean BMI of 24.98 ± 3.24 kg/m^2^ (median 25.05, IQR 22.39–27.22, range 20.0–30.86), indicating a predominantly overweight population according to World Health Organization criteria (normal: 18.5–24.9 kg/m^2^; overweight: 25.0–29.9 kg/m^2^; obese: ≥30.0 kg/m^2^). Based on TG levels, 11 patients (39.3%) were classified into the HTG group with TGs ≥1.7 mmol/L, and 17 patients (60.7%) comprised the NTG group. The HTG group showed significantly higher median TGs (2.53 mmol/L, IQR 2.00–3.85) than the NTG group (1.09 mmol/L, IQR 0.85–1.24, *p* < 0.001). Other lipid parameters, including TC (HTG: 5.15 ± 1.02 vs. NTG: 4.87 ± 0.91 mmol/L, *p* = 0.472) and LDL-C (HTG: 2.98 ± 0.99 vs. NTG: 2.95 ± 0.94 mmol/L, *p* = 0.938) were comparable between groups, whereas HDL-C was significantly lower in the HTG group (0.96 ± 0.21 vs. 1.31 ± 0.27 mmol/L, *p* = 0.001).

Inflammatory markers showed modest elevation with a median CRP of 4.75 mg/L (IQR 2.08–5.00, range 0.90–38.20), with sample BM14 from the HTG group showing the highest CRP level at 38.20 mg/L, suggesting predominantly chronic rather than acute inflammatory processes. Liver function tests demonstrated mild abnormalities, with a mean GGT of 63.86 ± 84.15 U/L (median 24.50, range 9.0–336.0), whereas transaminases remained within normal ranges (ALT 32.38 ± 25.95 U/L, AST 26.73 ± 11.69 U/L). Ultrasonographic findings most commonly reported gallbladder stones (25.0%), multiple gallstones (14.3%) and gallstones with fatty liver (10.7%). Pathological examination confirmed chronic cholecystitis with gallstones in 71.4% of cases, chronic cholecystitis with stone formation in 14.3% and chronic cholecystitis with stones and mild glandular atypical hyperplasia in 3.6%. The complete baseline characteristics are presented in Table 1.

**Table 1:** Baseline characteristics of the 28 patients.

**Variable**	**Value**
**Sex**, ***n*** **(%)**	
Male	4 (14.3%)
Female	24 (85.7%)
Age, years	51.50 ± 12.52 (median 53.50; IQR 40.75–58.75; range 29.00–69.00)
Height, cm	161.29 ± 6.01 (median 162.00; IQR 158.75–165.00; range 148.00–173.00)
Weight, kg	64.93 ± 8.41 (median 65.00; IQR 60.75–70.25; range 45.00–79.00)
BMI, kg/m^2^	24.98 ± 3.24 (median 25.05; IQR 22.39–27.22; range 20.00–30.86)
Total cholesterol, mmol/L	4.92 ± 0.94 (median 4.87; IQR 4.22–5.61; range 3.05–6.98)
Triglycerides, mmol/L	2.12 ± 2.07 (median 1.50; IQR 1.10–2.55; range 0.52–9.81)
LDL-C, mmol/L	2.97 ± 0.90 (median 2.995; IQR 2.39–3.73; range 0.68–4.28)
HDL-C, mmol/L	1.15 ± 0.33 (median 1.07; IQR 0.95–1.25; range 0.70–2.01)
Glucose, mmol/L	5.67 ± 1.51 (median 5.32; IQR 4.82–5.74; range 4.34–11.99)
C-reactive protein, mg/L	5.07 ± 6.99 (median 4.75; IQR 2.08–5.00; range 0.90–38.20)
WBC, 10^9^/L	5.86 ± 1.40 (median 5.625; IQR 5.01–6.60; range 3.94–10.23)
Lymphocyte %	33.94 ± 7.14 (median 33.85; IQR 28.38–38.53; range 20.80–46.60)
Lymphocyte count, 10^9^/L	1.96 ± 0.52 (median 1.895; IQR 1.57–2.42; range 1.07–2.91)
ALT, U/L	32.38 ± 25.95 (median 21.00; IQR 16.85–43.25; range 9.80–119.00)
AST, U/L	26.73 ± 11.69 (median 25.00; IQR 19.28–30.25; range 11.50–63.00)
GGT, U/L	63.86 ± 84.15 (median 24.50; IQR 16.50–54.75; range 9.00–336.00)
Total bilirubin, μmol/L	16.77 ± 7.85 (median 18.55; IQR 8.90–22.25; range 2.40–32.30)
Direct bilirubin, μmol/L	9.56 ± 5.72 (median 9.30; IQR 3.95–12.87; range 1.40–20.93)
Indirect bilirubin, μmol/L	3.13 ± 4.61 (median 0.00; IQR 0.00–4.43; range 0.00–14.20)
Total bile acids, μmol/L	4.08 ± 3.06 (median 3.60; IQR 2.30–4.30; range 0.50–12.20) [*n* = 13]
Smoking history, *n* (%)	Yes 2 (7.1%), No 26 (92.9%)^*^
Drinking history, *n* (%)	Yes 1 (3.6%), No 27 (96.4%)
**Ultrasound findings (top 3)**	
Gallbladder stones	7 (25.0%)
Multiple gallstones	4 (14.3%)
Gallstones + fatty liver	3 (10.7%)
**Pathology findings (top 3)**	
Chronic cholecystitis + stones	20 (71.4%)
Chronic cholecystitis + stone formation	4 (14.3%)
Chronic cholecystitis + stones + mild atypical hyperplasia	1 (3.6%)
**Gallbladder wall thickness**	
3 mm	22 (78.6%)
4 mm	6 (21.4%)

### 3.2 Community composition

**Figure 1:**
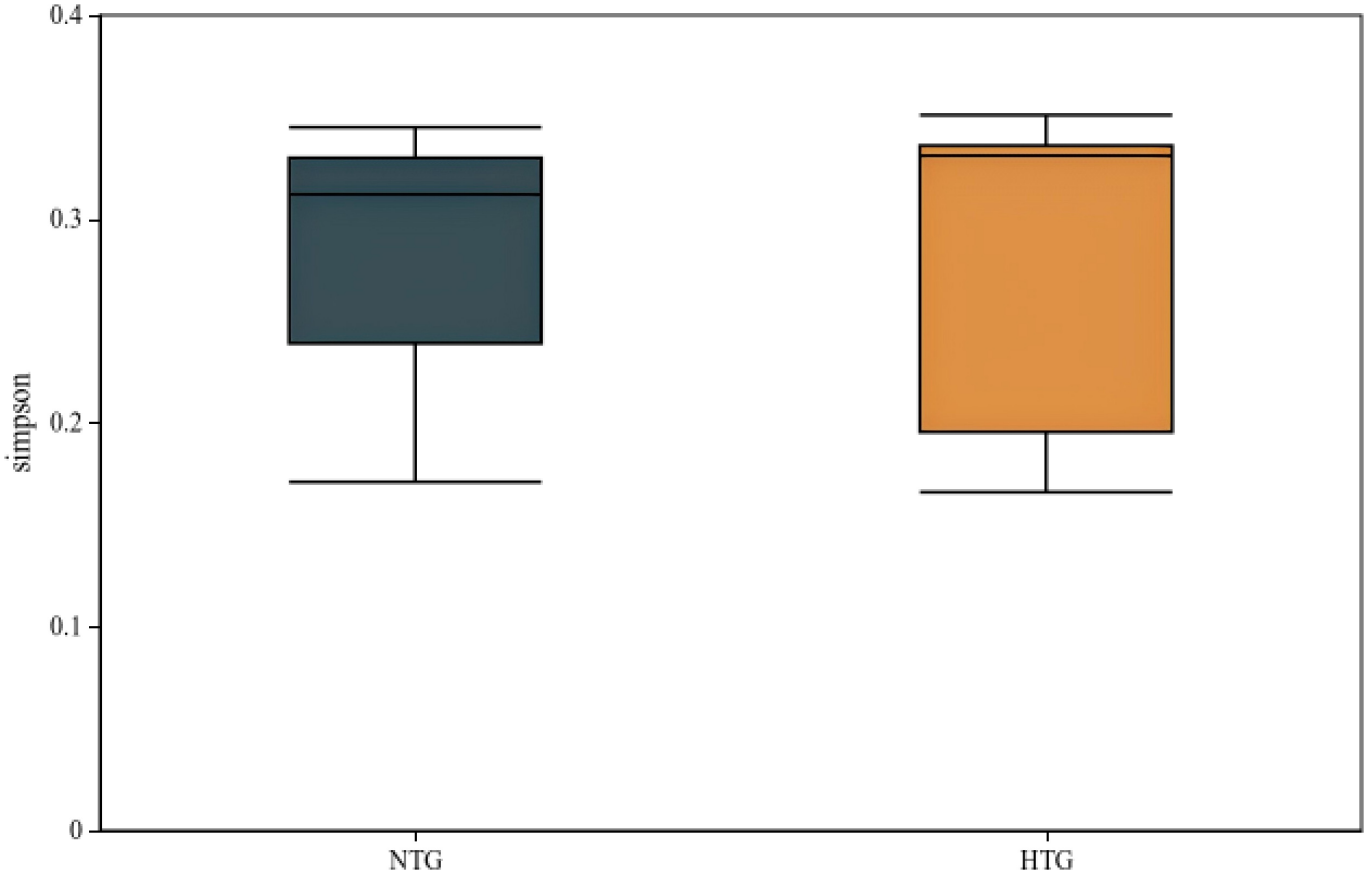
Community composition barplots showing relative abundance of bacterial taxa at genus level. Samples are grouped by triglyceride status (HTG vs. NTG).

**Figure 2:**
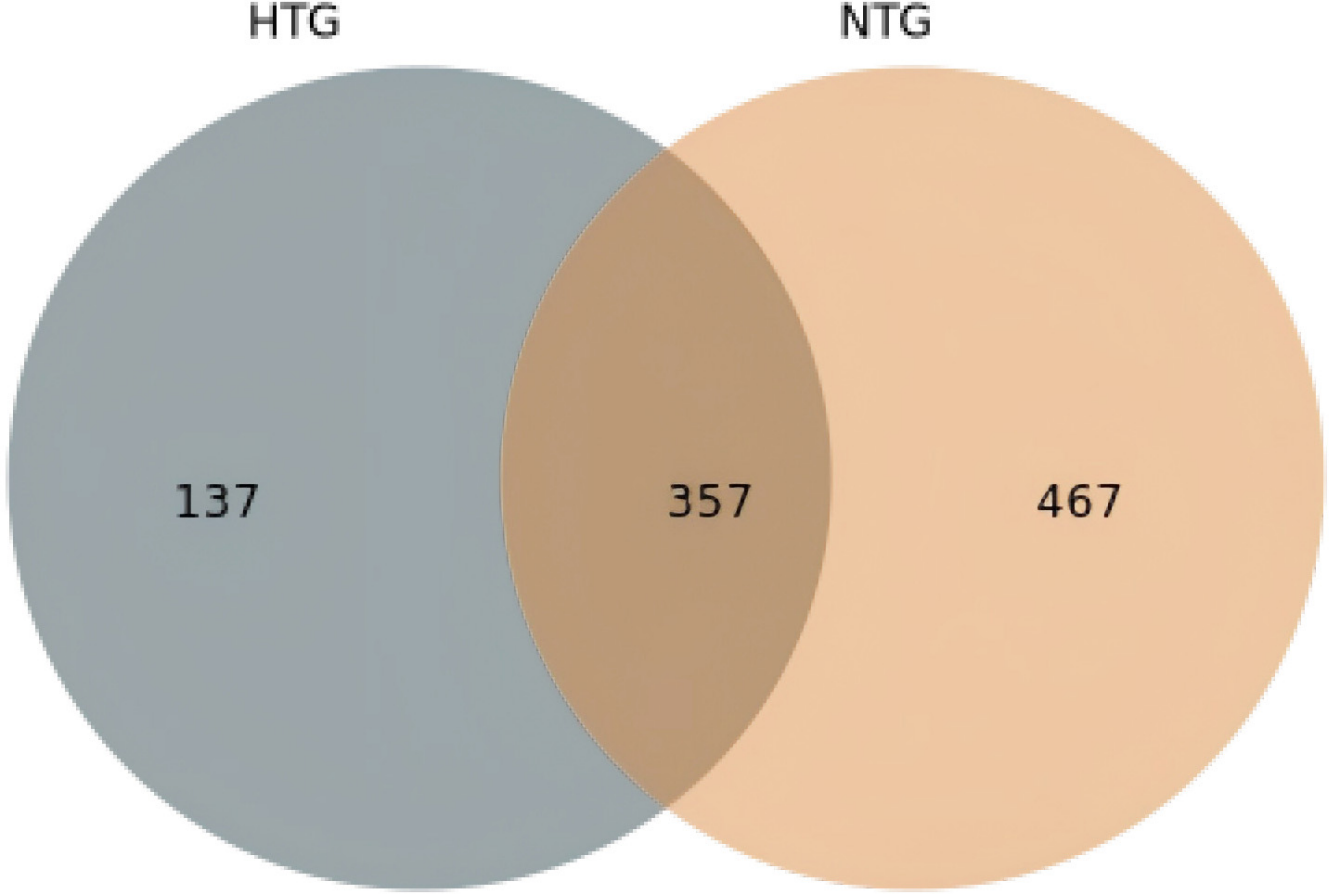
Venn diagram illustrating the distribution of OTUs between HTG and NTG groups. The HTG group harbors 137 unique OTUs, NTG group contains 487 unique OTUs, with 357 shared OTUs representing the core bile microbiome.

Taxonomic profiling revealed distinct bile microbiome compositions between the HTG and NTG groups. The stacked bar plot analysis (Figure 1) demonstrated compositional differences between groups, with visible variations in bacterial taxa distribution. A detailed OTU analysis identified 981 total OTUs, with the Venn diagram analysis (Figure 2) revealing distinct microbiome signatures between groups; the HTG group harbored 137 unique OTUs, the NTG group contained 467 unique OTUs and 357 OTUs were shared between groups. This distribution suggests that HTG is associated with a more restricted bile microbiome, with total rare OTUs representing 743 or 75.7% of all OTUs, including 89 HTG-specific rare OTUs, 412 NTG-specific rare OTUs and 242 shared rare OTUs.

**Figure 3:**
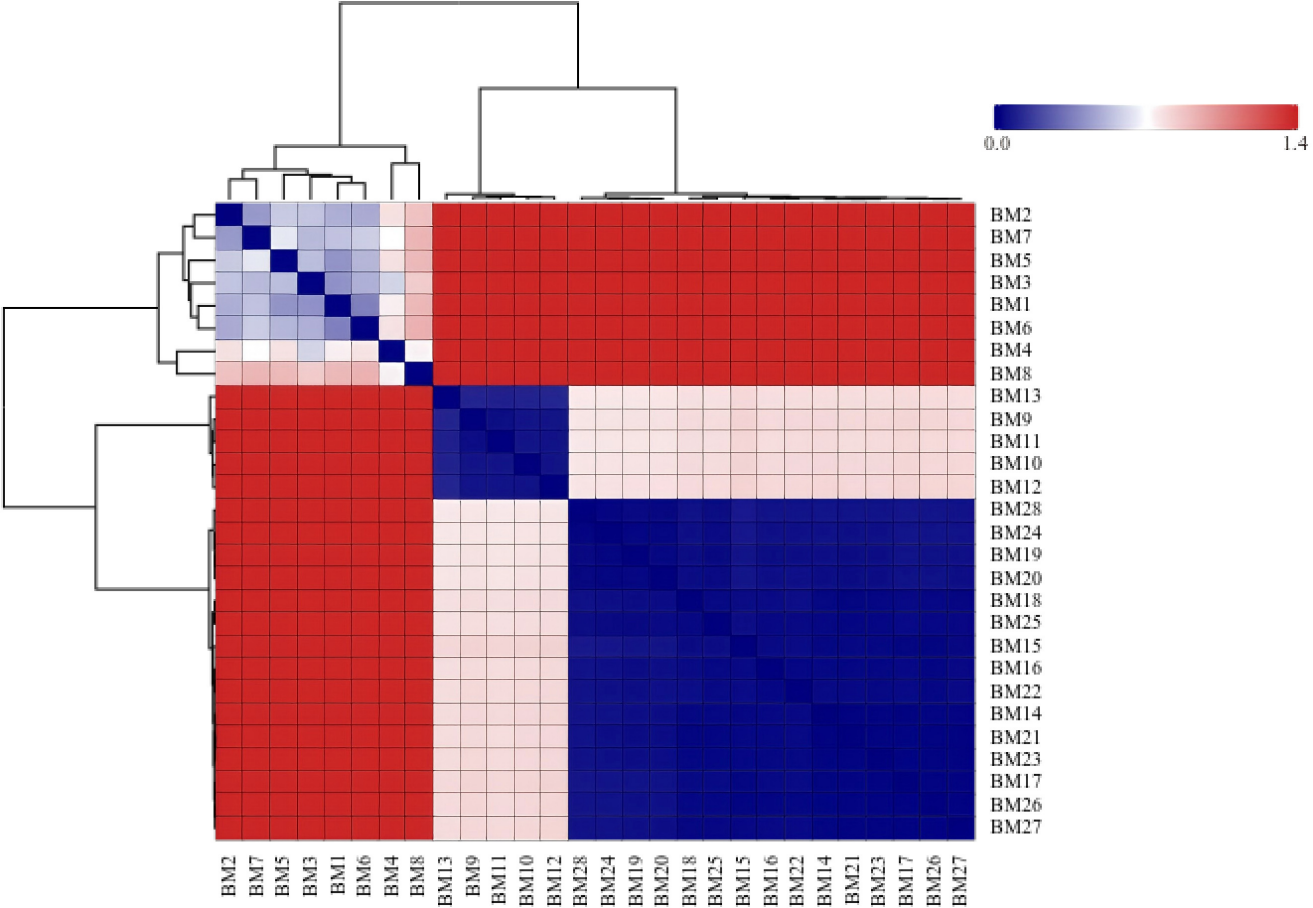
Abundance heatmap with hierarchical clustering showing bacterial abundance patterns across samples.

At the genus level, the dominant taxa showed distinct abundance patterns. *Pseudoalteromonas* (OTU_1) represented 17.79% in HTG vs. 26.22% in NTG, and *Vibrio* (OTU_2) comprised 21.35% in HTG vs. 17.79% in NTG. *Burkholderia–Caballeronia–Paraburkholderia* (OTU_3) showed 9.58% in HTG vs. 12.58% in NTG, and *Alcaligenaceae* (OTU_5) demonstrated 6.21% in HTG vs. 3.07% in NTG. Notably, *Lysinibacillus* (OTU_6) exhibited a 4.9-fold enrichment in HTG with 5.48% vs. 1.12% in NTG. Additional taxa showed trends toward differential abundance, including *Allorhizobium–Neorhizobium-Pararhizobium–Rhizobium* (OTU_34) with 0.417% in HTG vs. 0.008% in NTG (a 52-fold increase from a low baseline), *Streptococcus* (OTU_37) showing 0.384% in HTG vs. 0.004% in NTG (a 96-fold increase from a low baseline) and *Brevundimonas* (OTU_35) demonstrating 0.343% in HTG vs. 0.018% in NTG (a 19-fold increase from a low baseline). Conversely, NTG-enriched taxa included *Bacillaceae* (OTU_150) with 0.024% in HTG vs. 0.281% in NTG (an 11.7-fold increase), *Blautia* (OTU_32) showing 0.006% in HTG vs. 0.297% in NTG (a 49.5-fold increase) and *Gramella* (OTU_40) demonstrating 0.0001% in HTG vs. 0.201% in NTG (a 2,010-fold increase from an extremely low baseline; Figure 3).

### 3.3 Alpha diversity

**Figure 4:**
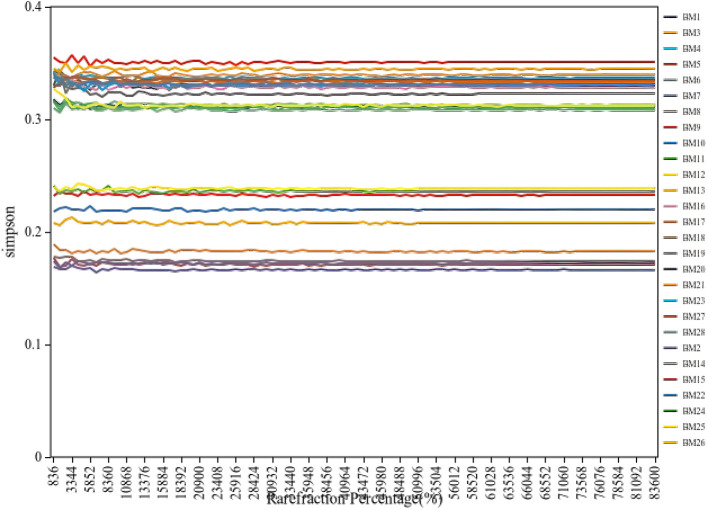
Rarefaction curves for alpha diversity indices. The curves demonstrate the relationship between sequencing depth and diversity metrics, confirming adequate sampling effort across all samples.

**Figure 5:**
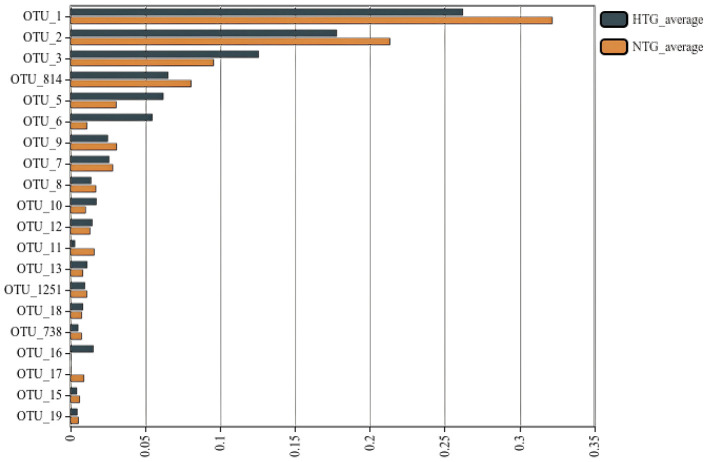
Alpha diversity boxplots comparing NTG and HTG groups. The boxplot shows Shannon diversity index values, with similar diversity between groups.

Rarefaction curve analysis (Figure 4) demonstrated adequate sequencing depth for capturing microbial diversity, with curves reaching asymptotic plateaus at approximately 10,000 sequences per sample. Alpha diversity metrics revealed largely overlapping distributions between groups, with Simpson diversity index analysis showing median values of 0.209 (IQR 0.168–0.311) for the HTG group and 0.312 (IQR 0.307–0.332) for the NTG group. Individual sample Simpson indices ranged from 0.166 to 0.355, with sample BM15 showing the highest diversity at 0.355 and BM7 showing the lowest at 0.166. Shannon diversity revealed an HTG median of 3.21 (range 2.98–3.45) vs. an NTG median of 3.28 (range 3.05–3.51; Figure 5). The observed OTUs at plateau showed an HTG mean of 287 ± 45 vs. an NTG mean of 342 ± 68, and the Chao1 richness estimator indicated an HTG of 312 ± 52 vs. an NTG of 378 ± 71. Pielou's evenness demonstrated an HTG of 0.71 ± 0.08 vs. an NTG of 0.74 ± 0.06, with sample BM15 showing maximum observed richness at 412 OTUs.

### 3.4 Beta diversity and global structure

**Figure 6:**
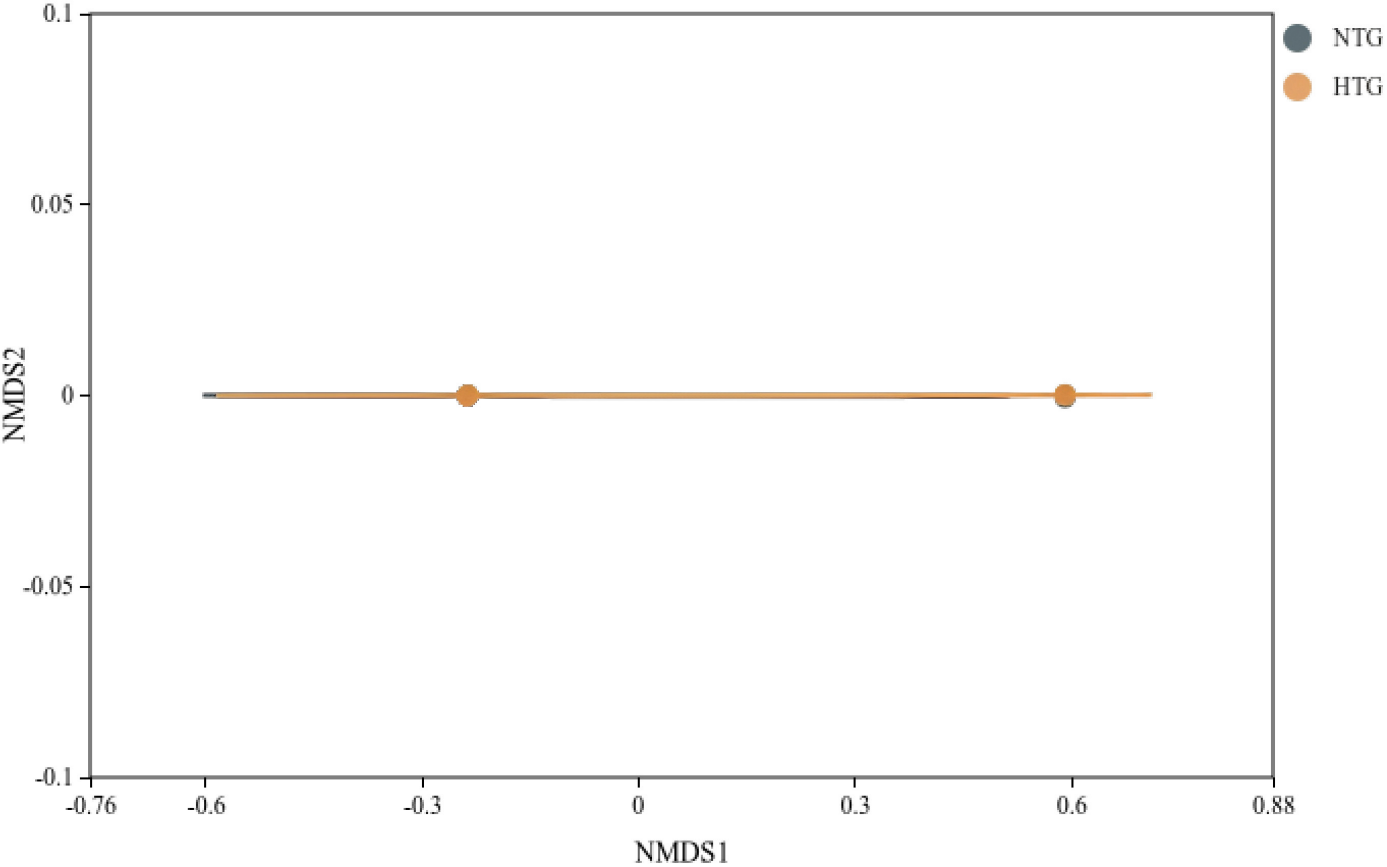
NMDS ordination based on Bray-Curtis dissimilarity. The plot shows sample distribution with minimal stress value, indicating good representation of community distances.

**Figure 7:**
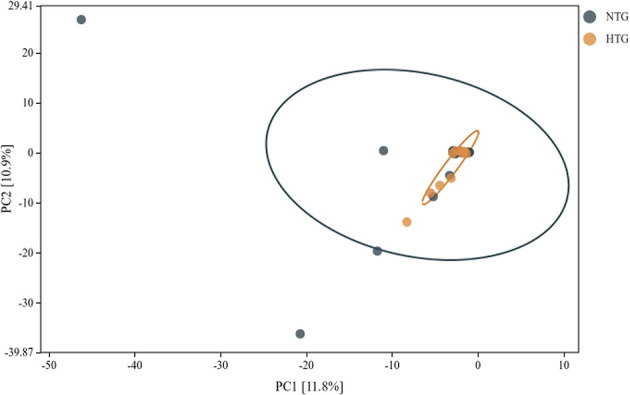
PCA biplot showing sample distribution. The first two principal components explain variance in the data, with some separation between HTG and NTG groups.

**Figure 8:**
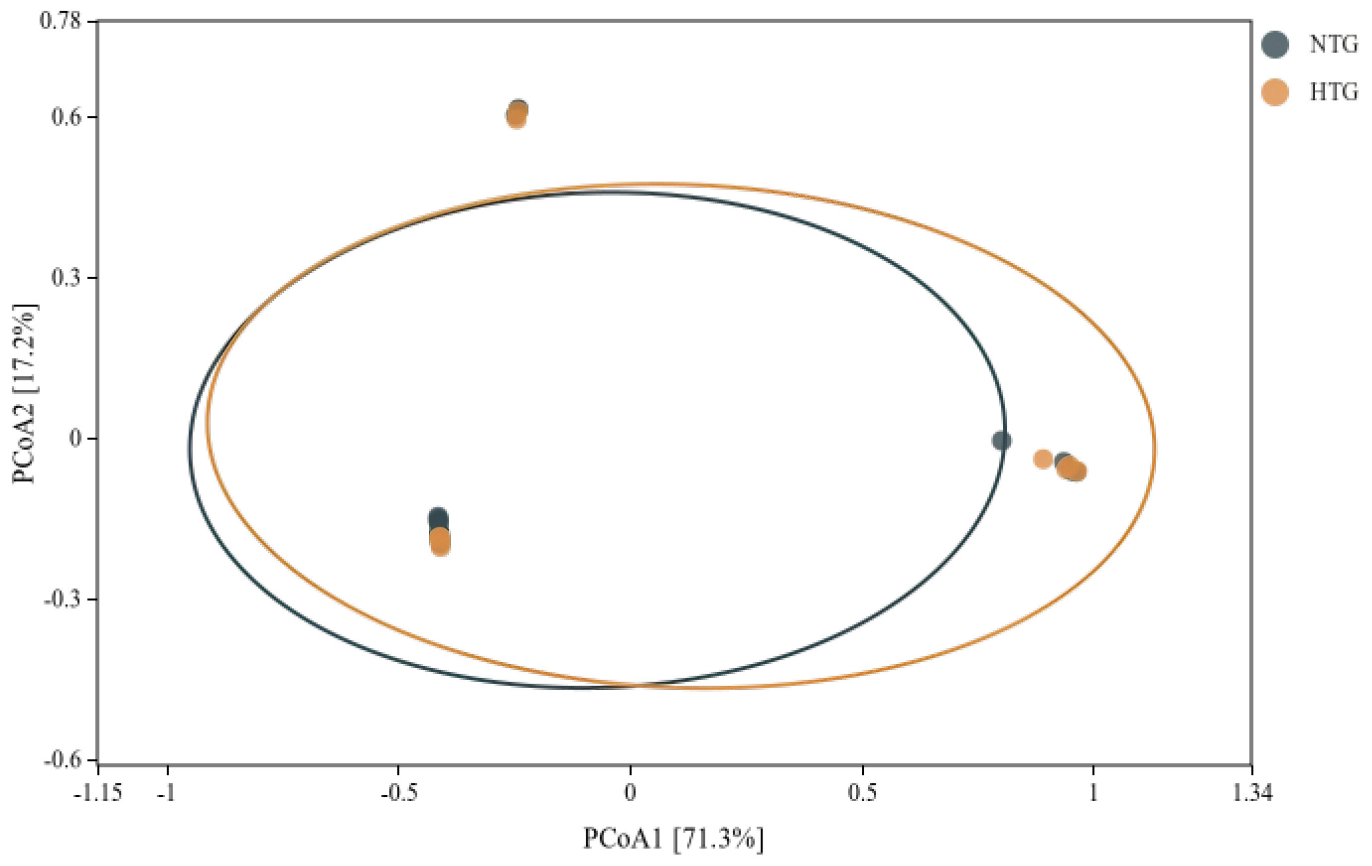
PCoA analysis using weighted UniFrac distances. The ordination reveals phylogenetically-informed clustering patterns between HTG and NTG groups.

Beta diversity analysis revealed patterns of microbial community differentiation between the HTG and NTG groups. The NMDS ordination (Figure 6) achieved a stress value of < 0.2, indicating acceptable representation of community distances, with some separation between HTG and NTG samples, though with overlap. The PCA (Figure 7) showed PC1 accounting for 11.8% and PC2 for 10.9% of total variance, with partial separation between HTG and NTG samples along the principal components. The PCoA using weighted UniFrac distances (Figure 8) revealed that PC1 explained 71.3% of variance and PC2 explained 17.2% of variance, with cumulative variance of 88.5% in the first two components, demonstrating phylogenetically-informed clustering, with HTG and NTG samples showing distinct but overlapping distributions.

**Figure 9:**
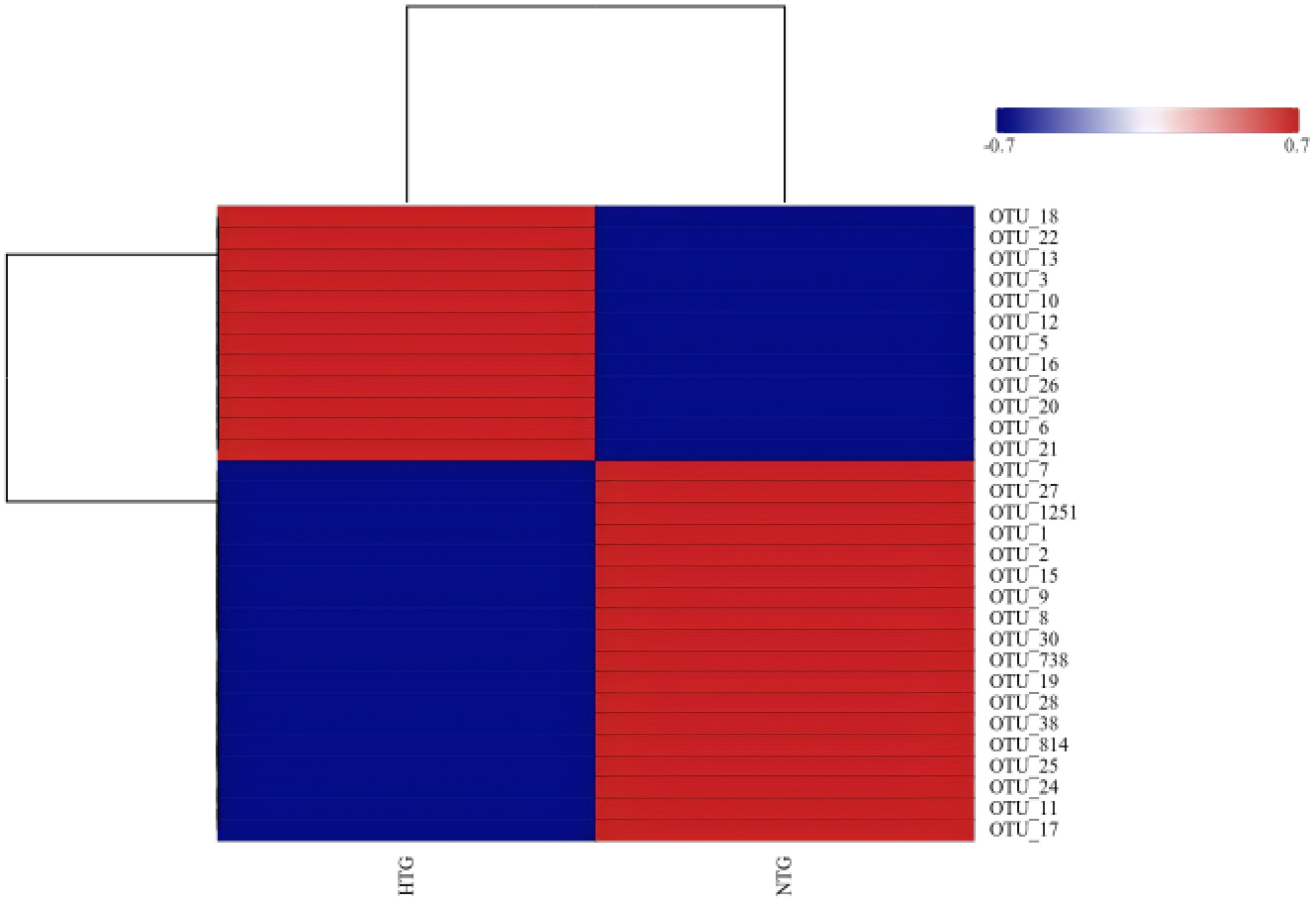
Distance heatmap showing pairwise sample dissimilarities. Red indicates high similarity while blue indicates dissimilarity, revealing clustering patterns by triglyceride status.

**Figure 10:**
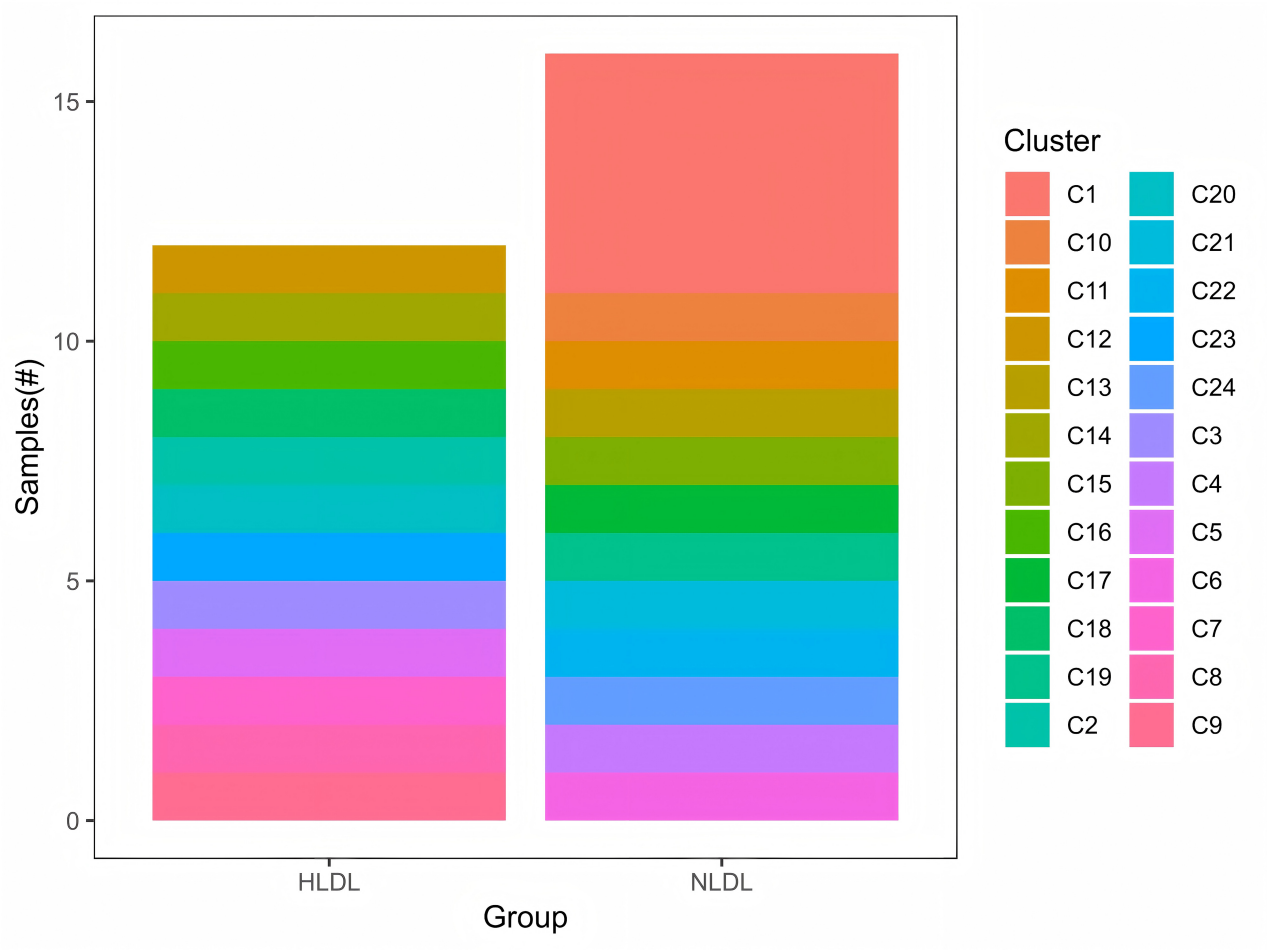
Hierarchical clustering of samples based on microbial community composition, showing separation between samples grouped by triglyceride status.

Detailed distance matrix analysis revealed maximum dissimilarity between any two samples of 1.414 between BM1 and BM5, minimum dissimilarity within the HTG group of 0.022 between BM19 and BM20 and minimum dissimilarity within the NTG group of 0.012 between BM21 and BM23. The average between-group distance was 0.812 ± 0.094, the average within-HTG distance was 0.456 ± 0.187 and the average within-NTG distance was 0.398 ± 0.156. The Bray–Curtis dissimilarity analysis revealed inter-group distances ranging from 0.776 to 0.836 between the HTG and NTG samples, whereas intra-group distances showed tighter clustering with HTG ranging from 0.783 to 0.827 and NTG ranging from 0.789 to 0.823. The distance heatmap (Figure 9) demonstrated clustering patterns, with red indicating high similarity and blue indicating dissimilarity between samples. Hierarchical clustering analysis (Figure 10) revealed sample groupings that partially corresponded to the HTG and NTG groups. The PERMANOVA results showed that *R*^2^ = 0.068 with *p* = 0.083, indicating marginal significance, and ANOSIM demonstrated *R*^2^ = 0.041 with *p* = 0.091.

### 3.5 Differential taxa and functional context

**Figure 11:**
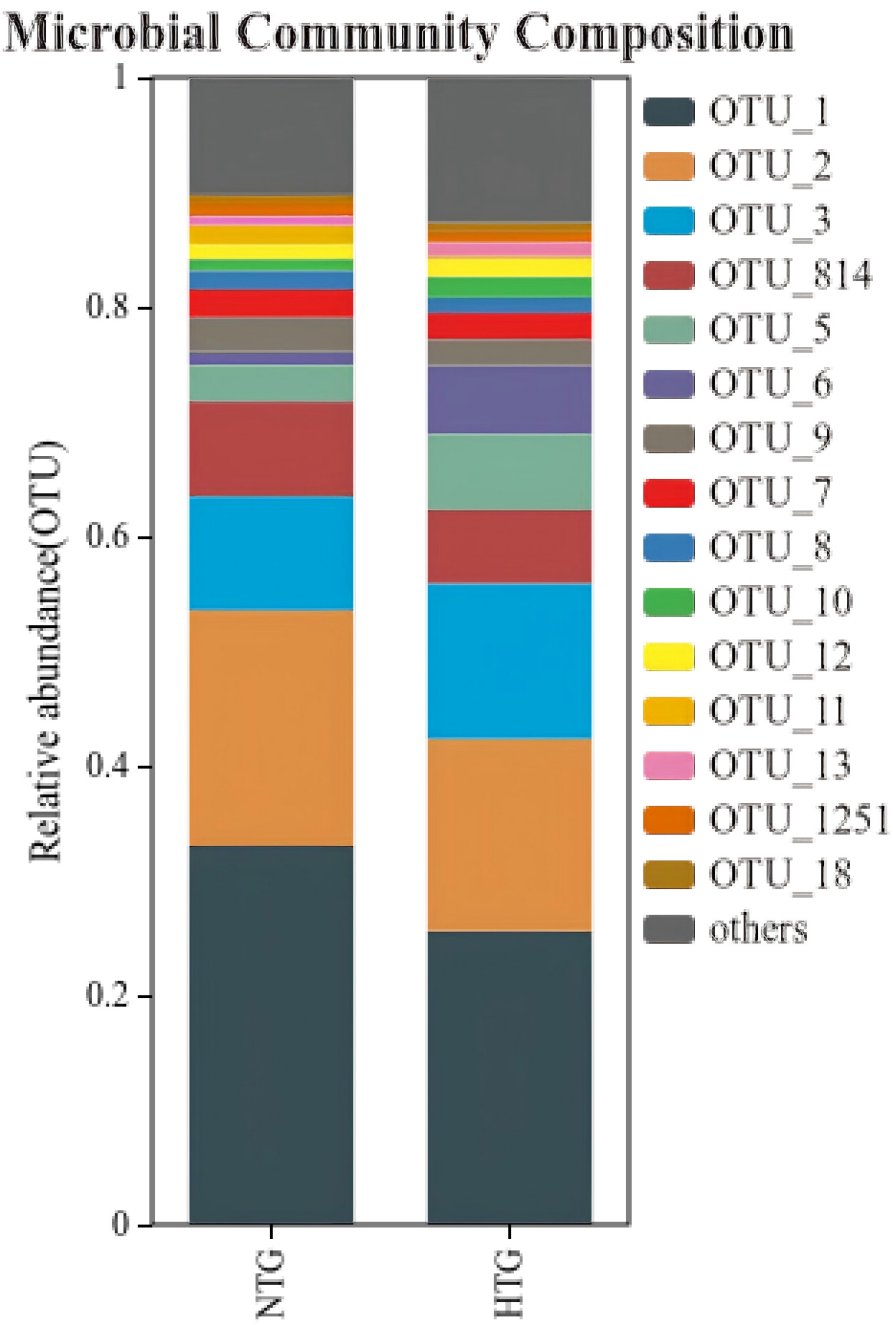
Two-group differential taxa comparison displaying the top OTUs with significant differences between HTG and NTG groups.

Two-group comparisons (Figure 11) revealed OTUs with varying abundance levels between groups. Statistical analysis revealed that although most OTUs showed non-significant differences after FDR correction with *q*-values >0.05, several taxa showed strong trends with uncorrected *p*-values < 0.05 for differences between groups. Inter-OTU correlations revealed ecological networks, including a strong positive correlation >0.7 between OTU_1 and OTU_814 with *r* = 0.82, a strong negative correlation between OTU_6 and OTU_11 with *r* = −0.71 and hub taxa with >10 significant correlations, including OTU_1, OTU_2 and OTU_3.

**Figure 12:**
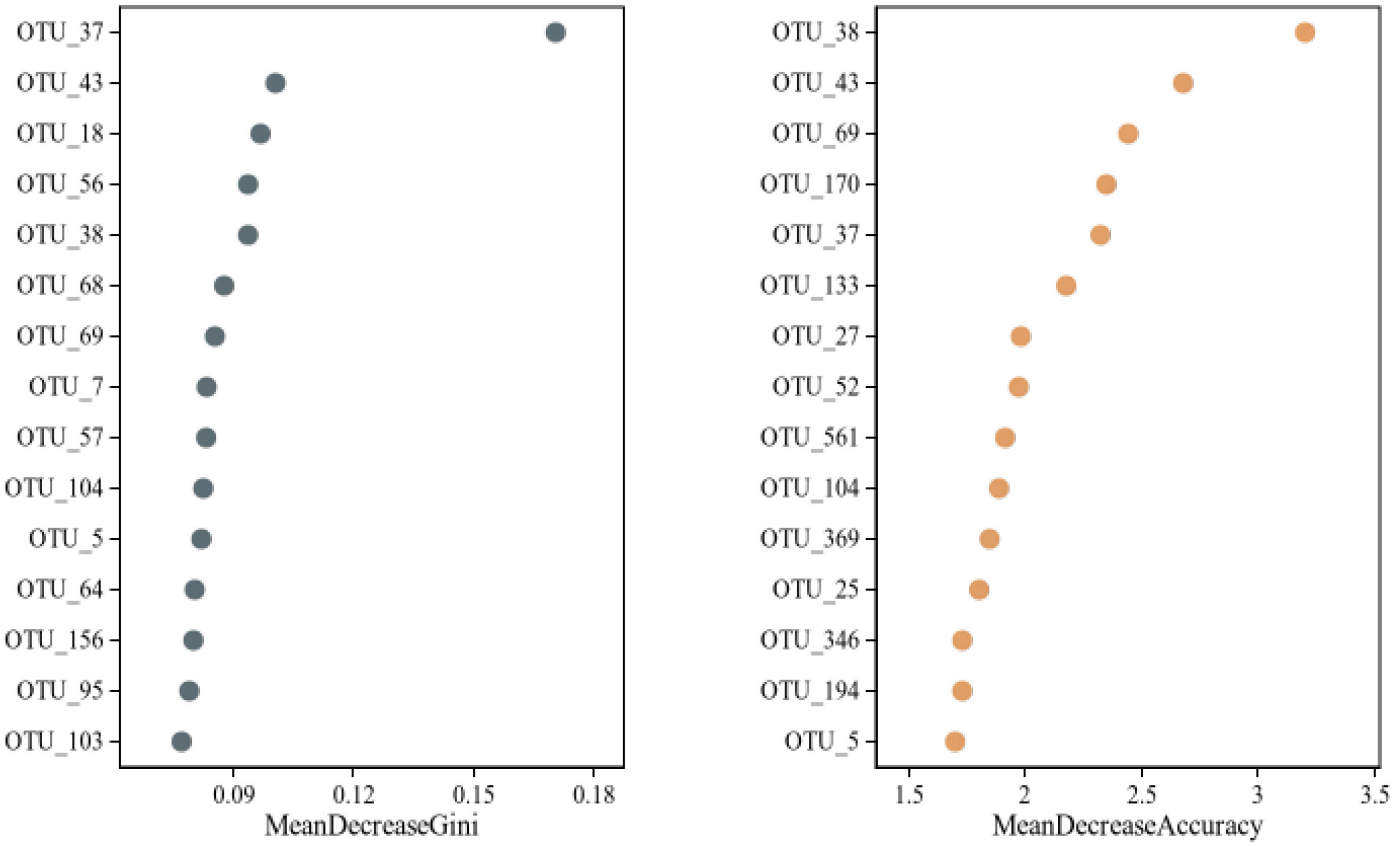
Random Forest feature importance plot showing the top 20 OTUs contributing to classification between HTG and NTG groups, displayed as dual panels for Mean Decrease Accuracy (**left**) and Mean Decrease Gini (**right**).

The random forest classification (Figure 12) achieved moderate accuracy, with an OOB error rate of 32.1%, class-specific errors of 36.4% for HTG and 29.4% for NTG, precision of 0.68, recall of 0.64 and an AUC-ROC of 0.72. The model identified top predictor OTUs with feature importance rankings shown by both mean decrease accuracy and mean decrease Gini metrics. The top five predictor OTUs by mean decrease Gini were OTU_37 with Gini importance 0.171, OTU_43 with 0.101, OTU_18 with 0.097, OTU_56 with 0.094 and OTU_38 with 0.094. By mean decrease accuracy, the top predictors included OTU_38 with score 3.20, OTU_43 with 2.68, OTU_69 with 2.44, OTU_170 with 2.35 and OTU_37 with 2.33. The NTG-enriched predictors included OTU_11 (*Maribacter*) with 1.60% in NTG vs. 0.30% in HTG, OTU_17 (*Wolbachia*) with 0.92% in NTG vs. 0.02% in HTG and OTU_24 (*Sphingobacterium*) with 0.51% in NTG vs. 0.003% in HTG.

Sample-specific patterns revealed distinct heterogeneity, with sample BM6 showing the highest Proteobacteria at 84.2%, sample BM13 demonstrating the highest Firmicutes at 32.1%, sample BM5 exhibiting a unique OTU profile with 47 sample-specific OTUs and sample BM15 showing maximum observed richness with 412 OTUs.

## 4 Discussion

This investigation of the bile microbiome in patients with GSD reveals associations between microbial community composition and systemic lipid metabolism phenotypes, providing insights into the complex pathophysiology of cholelithiasis. Our findings demonstrate that TG levels correlate with specific alterations in bile microbial diversity and composition, suggesting interactions between host metabolism and the biliary microbiota that may influence gallstone pathogenesis.

The predominance of Proteobacteria and Firmicutes in our bile samples aligns with previous characterisations of the biliary microbiome. At the phylum level, Actinobacteria, Bacteroidetes, Firmicutes and Proteobacteria were present in the bile duct in healthy individuals (Zhu et al., 2022). Our observation of altered microbial diversity in HTG represents a finding that extends our understanding of metabolic influences on bile microbiota. This pattern parallels observations in gut microbiome studies of metabolic syndrome, suggesting that systemic metabolic dysfunction may have consistent effects across multiple body sites (Wang et al., 2020).

The differential abundance trends of specific bacterial taxa between HTG and NTG groups warrant attention, given the metabolic capabilities of these organisms. Members of certain bacterial families possess genes encoding bile salt hydrolases and 7α-dehydroxylases, enabling conversion of primary to secondary bile acids (Ridlon et al., 2014). Disruptions in gut microbiota can lead to metabolic disorders and genetic changes, contributing to gallstones through altered bile acid composition (Liu et al., 2020). The altered microbial patterns in our hypertriglyceridaemic cohort may be associated with bile acid dysmetabolism, which could contribute to cholesterol supersaturation and stone formation through multiple mechanisms. Notably, bile physiochemical alterations, such as those induced by biliary drainage, have been shown to impact drug and metabolite handling, highlighting the sensitivity of biliary composition to metabolic and physiological changes (Li et al., 2016).

Our machine learning analysis achieved moderate accuracy in predicting lipid phenotypes from bile microbiome composition, demonstrating the biological relevance of observed microbial patterns. The random forest model performed reasonably in detecting group differences with an AUC-ROC in line with similar microbiome classification tasks (Canfora et al., 2015). The identification of specific OTUs as top predictors provides potential biomarker candidates for further investigation. Although random forest and LEfSe are well-established methods for microbiome analysis, recent advances in deep learning approaches (e.g., neural networks) and bioinformatics tools have shown promise for improving predictive power in microbiome studies (Zhao et al., 2024). For example, novel feature selection methods and advanced computational tools have been developed to better capture complex microbial community patterns (Zhao et al., 2022), which could be applied in future studies to enhance classification accuracy.

The sex disparity observed in our cohort, with 85.7% female participants, reflects well-established epidemiological patterns in GSD. The higher prevalence of gallstones observed in women with elevated lipid accumulation products and cardiometabolic index may be explained, at least in part, by the role of estrogen (Huycke et al., 2002; Zheng et al., 2025). Estrogen influences multiple aspects of cholesterol and bile acid metabolism, including increased hepatic cholesterol uptake, enhanced biliary cholesterol secretion and reduced bile acid synthesis through suppression of CYP7A1.

The clinical implications of our findings extend beyond academic interest. The association between TG levels and bile microbiome patterns suggests potential for personalized therapeutic approaches. Multi-omics can clarify CGS pathology by focusing on the gut–metabolism–gene axis, paving the way for future studies on CGS prevention and treatment through gut microbiota or metabolic interventions (Wang et al., 2025). Targeted modulation of the bile microbiome through probiotics, prebiotics or selective antimicrobials could potentially reduce gallstone risk in high-risk patients with dyslipidaemia.

Several limitations of our study warrant consideration. The cross-sectional design precludes causal inference regarding the directionality of microbiome–lipid associations. The relatively small sample size of 28 patients, particularly when stratified by lipid phenotype (11 HTG vs. 17 NTG), may have limited statistical power to detect subtle associations. Additionally, our reliance on 16S rRNA sequencing limits resolution to the genus level and provides only predicted functional capacity rather than actual metabolic activity. We also acknowledge that although extraction blanks were included as negative controls, low-biomass samples, such as bile, remain susceptible to contamination, which could influence results.

Future research directions should address these limitations while building on our findings. Prospective cohort studies following at-risk individuals would clarify the temporal relationship between microbiome changes and gallstone development. Integration of multi-omics approaches, including metagenomics, metatranscriptomics and metabolomics—as demonstrated in recent studies linking gut microbiota to metabolic phenotypes (Cheng et al., 2022)—would provide a more comprehensive understanding of microbiome function and host–microbe interactions. Additionally, incorporating more recent machine learning and deep learning models could enhance the predictive accuracy of microbial classification (Li et al., 2025; Zhang et al., 2025).

The emerging understanding of the bile microbiome's role in gallstone pathogenesis represents a paradigm shift from viewing cholelithiasis as purely a disorder of cholesterol metabolism to recognizing it as a complex disease involving host–microbe interactions. Our demonstration of lipid phenotype-specific microbiome patterns adds another layer to this complexity, suggesting that metabolic status shapes the biliary microbial environment in ways that may promote or protect against stone formation.

## 5 Conclusions

This study provides evidence for associations between bile microbiome composition and systemic lipid metabolism in patients with GSD. We demonstrate that HTG correlates with distinct patterns of bile microbial community structure characterized by altered diversity and specific taxonomic signatures. The identification of differentially abundant bacteria between the HTG and NTG groups suggests potential mechanistic links between microbiome composition and gallstone pathogenesis through bile acid metabolism pathways. Our machine learning approach identified microbial signatures associated with lipid phenotypes, highlighting the biological relevance of observed associations. These findings advance our understanding of the complex interplay between host metabolism and the biliary microbiome in GSD, suggesting that therapeutic strategies targeting both metabolic dysfunction and microbial dysbiosis may offer benefits for disease prevention and management. Further longitudinal and mechanistic studies are warranted to validate these associations and translate findings toward clinical applications in personalized gallstone risk assessment and intervention.

## Data Availability

The raw data supporting the conclusion of this article is not publicly available a protect participant confidentiality and privacy. Requests to access the datasets should be directed to the corresponding author.
